# mTOR Signaling: New Insights into Cancer, Cardiovascular Diseases, Diabetes and Aging

**DOI:** 10.3390/ijms241713628

**Published:** 2023-09-04

**Authors:** Anindita Das, Flávio Reis

**Affiliations:** 1 Division of Cardiology, Pauley Heart Center, Internal Medicine, Virginia Commonwealth University, Richmond, VA 23298, USA; 2 Centre for Innovative Biomedicine and Biotechnology (CIBB), University of Coimbra, 3004-504 Coimbra, Portugal; 3 Institute of Pharmacology & Experimental Therapeutics & Coimbra Institute for Clinical and Biomedical Research (iCBR), Faculty of Medicine, University of Coimbra, 3000-548 Coimbra, Portugal

The mechanistic/mammalian target of rapamycin (mTOR), a member of the phosphoinositide 3-kinase (PI3K) related kinase family, integrates intracellular and environmental cues that coordinate a diverse set of cellular/tissue functions, such as cell growth, proliferation, metabolism, autophagy, apoptosis, longevity, protein/lipid/nucleotide synthesis, and tissue regeneration and repair [1]. Although mTOR signaling is essential for proper cellular homeostasis, the aberrant activation of mTOR is potentially associated with a myriad of pathological outcomes, including different types of cancer, metabolic/cardiovascular/pulmonary diseases, and neurodegenerative disorders [2]. Considering the pathophysiological importance of mTOR signaling, we collected review articles, original research articles, and short communications in this Special Issue to advance our in-depth understanding of the mTOR-signaling network in different diseases for the development of novel mTOR-targeted therapeutic approaches.

mTOR is the core component of two structurally and functionally distinct protein complexes, mTOR complex 1 (mTORC1) and mTOR complex 2 (mTORC2). mTORC1 integrates various stimuli and signaling networks to promote anabolic cellular metabolism, but it blocks catabolic processes, such as autophagy, by regulating each step of the autophagy process, including induction, nucleation, elongation, and the formation of a double-membrane autophagosome, the formation of autolysosomes, and the recycling of autophagosome-sequestered substrates [3]. The dysregulation of the mTORC1-autophagy axis in muscles results in the development of diverse muscle diseases. Han et al. emphasized the essential role of balancing mTORC1 and autophagy in energy generation/consumption and macromolecule turnover processes for maintaining the physiological condition of skeletal muscles [4]. They also discussed about potential treatment options offered by restoring the balance between mTORC1 and autophagy to mitigate the progression of two muscle diseases, cancer cachexia and sarcopenia.

The PI3K/mTOR signaling pathway plays a pivotal role in regulating cellular homeostasis; therefore, the dysregulation of signaling is often involved in aging and in age-related pathologies, including cancer, cardiovascular diseases and diabetes, among others. Thus, understanding this highly non-linear system pathway—involving intricate regulatory mechanisms and crosstalk with neighboring—is mandatory to advancing biology and the development of new therapeutic approaches. Making use of the computational and experimental studies available in the literature, Ghomlaghi et al. provided a nice overview of the complex dynamic mechanistic network of PI3K/mTOR signaling, highlighting its interaction/interdependence with other major signaling pathways, competitive inhibition and epigenetic alterations properties, and associated interconnecting positive/negative regulatory loops (feed-back/feed-forward mechanisms) [5]. In this article, the authors highlighted the use of computational modelling to investigate such a complex network properties of the PI3K/mTOR signaling pathway, to integrate and process all the information from many other signaling pathways and finally design effective patient-specific therapeutic strategies to combat various diseases. The authors systematically reviewed the crosstalk between the PI3k/mTOR signaling and the cell-cycle signaling networks, as well as their links with nutrient sources: the NF-kB, Ras/ERK, and hippo/MST signaling pathways.

As second messengers, the intracellular levels of cyclic nucleotides, cAMP (cyclic adenosine monophosphate), and cGMP (cyclic guanosine monophosphate) control all major cellular signaling pathways in cell proliferation, cell cycle regulation, and metabolic functioning. cAMP and cGMP are produced by activated adenylyl-cyclase and guanylyl-cyclase, respectively, and degraded by phosphodiesterases (PDEs). cAMP/cGMP and their associated kinases, including protein kinase A (PKA) and protein kinase G (PKG), directly/indirectly regulate mTOR signaling by phosphorylating different components of mTOR complexes under various physiological and pathological conditions. In the review article by Shi and Collins, the recent findings about the regulation of the mTOR signaling network by cAMP and cGMP, as well as by their corresponding protein kinases, PKA and PKG, were briefed [6]. They also discussed the adverse effects of dysregulated mTOR signaling in different cardiometabolic diseases, specifically focusing on obesity, non-alcoholic fatty liver disease, and cardiac remodeling and potential strategies involving PKA/PKG activation or PDE inhibition to fight the disease. 

Despite the growing number of people suffering from a severe hearing impairment, hearing aids or cochlea implants are the most effective and common treatment options. Currently, no curative intervention has been clinically used to treat sensorineural hearing loss (SNHL) because of the lack of understanding about the potential mechanisms underlying SNHL. New pharmacological strategies have been proposed that can promote cell survival pathways in sensory hair cells. A growing number of studies have illustrated the potential regenerative benefits of using progenitor cells of the chochlea, which can stimulate the differentiation of these progenitor cells into the hair cells and neurons to alleviate hearing impairment [7]. A couple of studies evaluated the role of mTOR signaling in cochlear hair cell proliferation and regeneration, which seem to involve compensation for the loss of function of some key players in the process, including *c-myc* and Notch1 genes, phosphatase, and tensin homolog (*Pten*), as well as the RNA binding protein LIN28B, among other potential mechanisms and targets [8,9]. mTOR signaling, by governing cell growth and survival pathways, among others, is a promising therapeutic target. In this Special Issue, Cortada et al. reviewed the most recent findings regarding mTOR signaling in the inner ear and discussed its critical role in age-related hearing impairments and promising strategies for hearing restoration by regulating auditory sensory hair cell survival and death [10]. The authors systematically emphasized the differential roles of mTORC1 and mTORC2 in hearing impairment. They rigorously discussed the deleterious effects of the overactivation of mTORC1 and the beneficial role of mTORC2 in hair cells. The authors also highlighted open questions about the future perspectives of pharmacological interventions with mTORC1 inhibitors for hearing restoration, by preserving the hair cells and related neural structures, in association with the alteration of ROS and/or autophagy, among other pathways.

This Special Issue also features a few original research articles on the therapeutic potential of mTOR inhibitors in different pathological conditions. Several lines of evidence implicate that mTOR signaling is critical for physiologic wound healing and pathologic fibrogenesis [11]. Aberrant mTOR signaling influences the development of fibrotic disease by inducing fibroblast proliferation, TGF-β1-induced myofibroblast differentiation, the abnormal accumulation of extracellular matrix (ECM), and collagen production [12]. Due to the anti-proliferative property of rapamycin (mTOR inhibitor, mTORi), rapamycin-eluting stents prevent the neointimal formation of graft coronary arteries and reduce the incidence of restenosis following coronary angioplasty, which is highly effective in reducing the risk of coronary artery disease [13,14]. By implanting rapamycin-eluting biodegradable stents (DE stent) into rabbits with thermal-injury-induced urinary tract obstructions, Ho et al. investigated the effect of sustainable local treatment with mTORi on the ureteral tract stricture [15]. They revealed that mTORi-eluting biodegradable stents significantly alleviated the urinary tract obstruction after thermal injury by regulating several mediators/markers of fibrosis, i.e., type III collagen, α-SMA and TGF-β, and EMT (Epithelial–Mesenchymal Transition) signaling pathway markers, p-Smad/Smad and SNAI 1. This study suggested the potential beneficial effects of intra-ureteral rapamycin-eluting stents, which could alter ureteral fibrosis signaling pathway, and ultimately, ameliorate a ureteral stricture after injury. 

In the heart, glycogen synthase kinase-3 beta GSK3β has been shown to play a major role in cardiac ischemic injury and hypertrophy. The activity of GSK-3β is inversely regulated by phosphorylation at serine-9 (S9) residue via AKT, which has been shown to regulate hypertrophic cardiomyocyte growth and viability and protects hearts against myocardial ischemia/reperfusion injuries [16,17]. However, the biological function of other GSK3β phosphorylation sites in cardiomyocytes, including those at serine 389 (S389), remains to be elucidated. In addition to Akt, the mTOR-p70S6K pathway also leads to the phosphorylation and inactivation of GSK3β. Reciprocally, GSK3β can activate mTOR through TSC1/2. To clarify the significance of the phosphorylation of GSK3 at S389 in the myocardium, Vainio et al. assessed GSK3β S389 phosphorylation in diseased hearts from mouse and human patients [18]. They noticed an elevated level of phosphorylation of GSK3β at S389 in the left ventricle of the patients with end-stage dilated or ischemic cardiomyopathy. The phosphorylation of GSK3β was also stimulated in the hearts of mice following thoracic aortic constriction-induced heart failure. The rat cardiomyocytes viability was reduced after the overexpression of either GSK3β S9A or S389A following hypoxia–reoxygenation injury, which was further reduced by the overexpression of the double GSK3β mutant (S9A/S389A). Moreover, both basal and agonist-induced hypertrophic cardiomyocyte growth were augmented following the overexpression of GSK3β S389A or GSK3β S9A/S389A. In this elegant study, the authors established that GSK3β S389A mutation positively regulated the activation of mTORC1 signaling, which led to cardiomyocyte hypertrophic growth. 

Patients with diabetes have a poor prognosis after myocardial infarction (MI), which leads to the development of heart failure with a higher risk of mortality [19] due to aggravated pathological myocardial remodeling and inflammation with concomitant cardiac dysfunction [20]. The persistent hyperactivation of mTOR signaling in diabetes exacerbates post-ischemic myocardial injuries due to accelerated cardiomyocyte death with cardiac remodeling and inflammatory responses. Our prior studies reported that a rapamycin treatment at the onset of reperfusion reduces the myocardial infarct size in diabetic mice and rabbits by inhibiting mTORC1, while restoring mTORC2 [21,22]. In this Special Issue, Samidurai et al. provides compelling evidence that acute reperfusion therapy with rapamycin restores post-MI cardiac function with the alleviation of adverse post-infarct myocardial remodeling and inflammation, which is indicated by suppressing several fibrosis markers (TGF-β, Galectin-3, MYH, and p-SMAD) and preventing NLRP3-inflammasome formation in diabetic rabbits [23].

Endothelin-1 (ET-1), a potent vasoconstrictive peptide produced by the endothelial cells, stimulates cell proliferative, fibrosis, and inflammation through binding to the endothelin receptors, ETA-R and ETB-R, which are abundantly expressed in cardiac tissues. Several lines of evidence suggest that the ET-1 and ET-R expression levels are elevated in the plasma and hearts of diabetic patients, and endothelin receptor antagonists protect against diabetes-induced cardiac injuries [24,25,26]. A previous study showed that under high-level glucose (HG) conditions, ET-R expression is regulated via adenosine monophosphate-activated protein kinase (AMPK)/mTOR signaling pathways [27,28]. Although the impairment of ET-1 signaling and the mTOR pathway have been established in diabetic cardiomyopathies, the interplay between them under HG conditions remains to be elucidated, which was the goal of Pandey et al. [29]. They found that HG conditions significantly reduce the survival of H9c2 cardiomyoblasts with an increase in the ROS level and mitochondrial fragmentation, as well as the loss of the mitochondrial membrane potential (MMP). They noticed that the significant induction of expressions of ET-1/ETA-R/ETB-R as well as mTOR/Raptor/Rictor under HG conditions. All these adverse effects on cellular functions were neutralized by the pharmacological inhibition of either ETA-R alone by ambrisentan or ETA-R/ETB-R by bosentan or the partial blockage of the mTOR functions by silencing Raptor or Rictor. Collectively, this study established the significant contribution of mTOR signaling in ET-1-induced mitochondrial damage under diabetic conditions.

Preclinical studies and randomized clinical trials established that pharmacological inhibitors of mTOR counteract age-associated pathogenesis to extend the patient’s longevity. Employing artificial intelligence (AI)-assisted approaches, Vidovic et al. identified a potent mTOR inhibitor, TKA001, with low toxicity, favorable physicochemical properties, and a preferable ADMET (absorption, distribution, metabolism, excretion, and toxicity) profile from 1000 small molecules predicted to target mTOR [30]. They modeled TKA001 binding in silico via molecular docking and molecular dynamics, which could inhibit both mTORC1 and mTORC2 signaling. They demonstrated the anti-proliferative effects of TKA001 on human prostate, epithelial, and cervical cancer cells. They also confirmed the anti-aging effect of TKA001 by extending the lifespan of *Caenorhabditis elegans*.
